# Study on Mechanical Properties and Impact Energy Release Characteristics of Skeleton-Structured Al/Ni Reactive Materials

**DOI:** 10.3390/ma18040900

**Published:** 2025-02-19

**Authors:** Zhichao Sun, Yansong Yang, Rui Zhang, Lei Guo, Yuan He, Enliang Liu, Chuanting Wang, Yong He

**Affiliations:** 1 School of Mechanical Engineering, Nanjing University of Science and Technology, Nanjing 210094, China; sunzhichao198851@163.com (Z.S.); njlgyys2021@163.com (Y.Y.); guolei@njust.edu.cn (L.G.); heyuan@njust.edu.cn (Y.H.); 2 Southwest Technology and Engineering Research Institute, Chongqing 400039, China; 13896218928@163.com; 3 Inner Mongolia Power Machinery Research Institute, Hohhot 010010, China; liuenliang_003@yeah.net

**Keywords:** reactive material, skeleton-structured, aluminum/nickel, quasi-sealed chamber, impact energy release

## Abstract

Reactive materials can be employed to realize the integration of damage element kinetic energy and chemical energy damage, as well as strengthen the destruction ability of warheads. Among them, Al/Ni material has become a research hotspot because of its simple structure, easy process, and high reaction heat. In this paper, a skeleton-structured Al/Ni reactive material was successfully prepared. Additionally, both static and dynamic mechanical performance tests were conducted, along with impact experiments in a quasi-sealed chamber. Furthermore, numerical simulations of the mechanical properties of the materials were performed. The results show that the prepared reactive material has a compressive strength of 150 MPa and a tensile strength of 51 MPa, and the numerical simulation results are in good agreement with the experimental data. The impact experiments and product recovery analysis show that the material has a certain energy release ability, and the overpressure can attain 0.081 MPa at a velocity of 1370 m/s in an air atmosphere. However, the overpressure in all experiments under an argon atmosphere is less than 0.02 MPa, which proves that the main reaction under the impact condition is an oxidation reaction rather than a metal intermetallic reaction. The results of this paper provide theoretical support and a data basis for the design of three-dimensional skeletons of reactive materials and the structural optimization and improvement in mechanical properties and have certain guiding significance in the application of Al/Ni reactive materials.

## 1. Introduction

Recently, reactive materials have received extensive research attention. Reactive materials are a type of mixed material prepared with two or more types of materials, including intermetallic compounds, metal/polymer mixtures, thermite, amorphous alloys, etc. [1,2]. Under impact, reactive materials can undergo a violent combustion reaction to achieve double the damage of kinetic energy and chemical energy, significantly enhancing the damage ability of the damage element to the target [3]. At present, the most widely used materials include Al-PTFE, Zr-PTFE, and other metal-fluoro polymers, as well as zirconium-based amorphous alloys, high-entropy alloys, etc. [4,5,6,7,8,9]. Al/Ni material is a reactive material with an intermetallic compound reaction system that has a simple structure and a high reaction calorific value. It has been widely studied by scholars in the fields of preparation technology, mechanical properties, reaction mechanisms, and impact energy release characteristics [10,11,12,13,14].

Al/Ni reactive materials have a simple structure, can be easily elaborated due to facile access to raw materials, and can be processed in various ways, which mainly include accumulative roll bonding [15], electrodeposition [16], the arc melting method [17], powder pressing [18], etc. However, the materials obtained by the above molding methods all rely on the force between aluminum and nickel molecules, which cannot form a continuous overall structure and sufficient metallurgical combination, resulting in poor tensile impact properties. In order to expand the application scenarios of Al/Ni reactive materials, it is necessary to improve their mechanical properties; this is mainly achieved by improving the fabrication process, for example by adding tungsten fiber to play the role of a fiber reinforcement [19], using accumulative roll-bonding technology to prepare multi-layer metal [20,21], using the explosive consolidation method to improve the density of the powder mixture [22], or adding heavy metal elements to improve the mechanical properties of the Al-Ni system [23,24].

As a typical two-component composite material, the optimal design of this material can also be achieved by designing the matrix of a skeleton structure. In the preparation of Al/Ni reactive materials, a component material is selected to prepare the skeleton matrix, and the other component material is filled in the form of filling the skeleton matrix to form an Al/Ni reactive material with a skeleton structure matrix, which can significantly improve its mechanical properties. At present, for the skeleton matrix, interpenetrating-phase composites are commonly used. Compared with one-dimensional and two-dimensional composite materials, such as particle reinforcement and fiber reinforcement, three-dimensional network-reinforced composites have better mechanical properties, shock absorption properties, and thermal properties [25,26,27,28,29].

In this paper, an Al/Ni reactive material containing a nickel metal skeleton is prepared. The mechanical properties, failure behavior, and impact energy release characteristics of the skeleton-structured Al/Ni reactive material under different strain rates are studied by combining theoretical, experimental, and numerical simulation methods. During the material design process, a mesh skeleton structure was innovatively applied to the Al/Ni reactive material. Comparative experiments validated that the energy release behavior of this material mainly originates from the oxidation of metals rather than from intermetallic compound formation. This study provides a reference for the application of skeleton structures in reactive materials and establishes a foundation for investigating the energy release behavior of skeleton-structured reactive materials.

## 2. Material Preparation and Experimental Methods

### 2.1. Material Preparation

**Figure 1:**
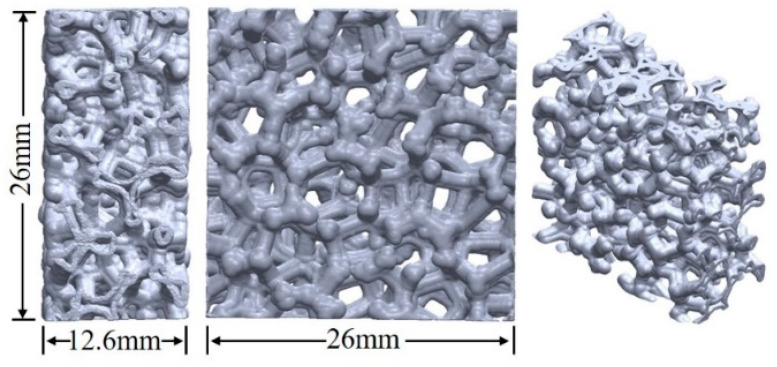
Local model of nickel skeleton.

In order to prepare the nickel skeleton matrix, polyurethane soft foam was used as the base material. The matrix was electrically treated with an alkaline electroless nickel plating system under the conditions of a current ranging from 60 A to 80 A and a temperature ranging from 40 °C to 50 °C. The nickel used was pure nickel from the Shanghai Hexinquan Metal Processing Plant, with a purity of over 99.99%. The nickel skeleton sample was prepared by repeated electroplating and heating treatment. A square was cut in the prepared nickel skeleton, and the nickel metal skeleton structure was scanned with CT scanning equipment (YXLON FF35CT, Comet Yxlon, Hamburg, Germany), and many sectional pictures were obtained. After filtering and reducing the noise in the images with MATLAB (R2019b) software (MathWorks, Natick, MA, USA), the threshold segmentation operation was carried out, and CT scan images with clear boundaries were obtained for subsequent research and the analysis of modeling and numerical simulation. The local model of the nickel skeleton established is shown in Figure 1. The relevant details and local characteristics of the nickel skeleton can be clearly seen from the model diagram, in which the diameter of the rod is about 1.24 mm, and the spacing of adjacent units is about 5 mm.

**Figure 2:**
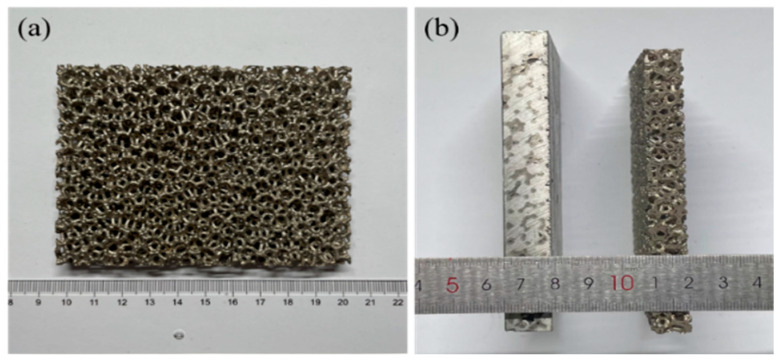
The prepared sample: (**a**) nickel skeleton; (**b**) comparison between the original skeleton and the sample after wire-cutting treatment.

As shown in Figure 2a, for the cut nickel skeleton, the aluminum liquid with the peroxide layer removed was poured into the nickel skeleton, and the molten aluminum was filled into the nickel metal skeleton by gravity. The aluminum used is commercially pure aluminum (Shandong Qianyu Aluminum Co., Ltd., Weifang, Shandong, China), with a purity of over 97%. After natural cooling, the ingot was subjected to wire-cutting treatment to obtain the skeleton-structured Al/Ni reactive material sample, as shown in Figure 2b. From the direction of reaction heat, when the molar ratio of aluminum to nickel is 3:2, the energy released by the aluminum–nickel reaction is the largest. The density of the Al/Ni material prepared by this ratio is 4.16 g/cm^3^, close to 91% of its theoretical density.

### 2.2. Mechanical Properties Test

**Figure 3:**
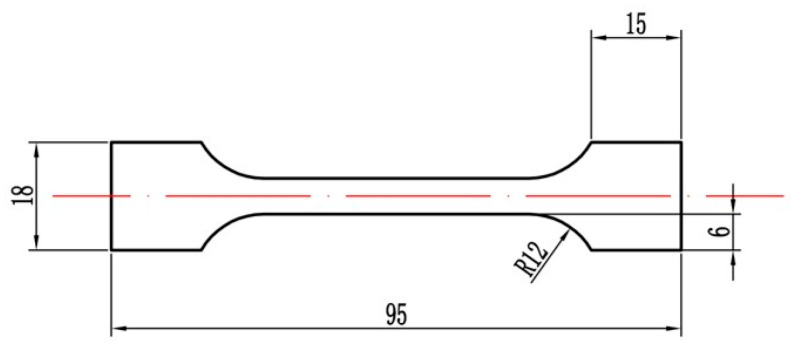
Size diagram of tensile specimen. (Units in mm).

In the test, a microcomputer-controlled electronic universal material testing machine (CSS-44300, Changchun Testing Machine Research Institute, Changchun, China) was used to carry out quasi-static compression tests at room temperature. The strain rate was 10^−3^ s^−1^, and the sample size was Φ10 mm × H20 mm. The specific size of the sample for the quasi-static tensile test is shown in Figure 3. The thickness is 6 mm, and the gauge length parallel to the loading direction is 45 mm. The strain rate was 10^−3^ s^−1^. The tensile sample was polished before the test to remove the surface oxide layer. To obtain the mechanical properties of the material under dynamic compression, split Hopkinson pressure bar (SHPB) experiments were carried out under different strain rates. The sample size was Φ10 mm × H5 mm. The experiments were repeated more than three times to reduce the influence of error. The tests were all conducted at room temperature.

### 2.3. Microstructure Characterization

The prepared skeleton-structured Al/Ni reactive material was polished to remove the oxide layer on the surface of the sample. The microstructure was analyzed by scanning electron microscopy (JSM-IT500HR, JEOL Ltd., Tokyo, Japan) under a high voltage of 20 kV, and the height of the camera was 10 mm from the surface of the sample. The energy dispersive spectrometer (EDS) device was used to determine the element composition of the sample. The sample size was Φ10 mm × H9 mm. The samples were recovered after the alignment static test and SHPB test, and the fracture surface was observed and analyzed by SEM.

### 2.4. Quasi-Sealed Chamber Experiment

The prepared skeleton-structured Al/Ni reactive material was cut to obtain the Φ10 mm × H9 mm cylindrical sample required for the impact experiment. Meanwhile, the cold-pressed Al/Ni reactive material was prepared and compared with the skeleton-structured Al-Ni reactive material. The specific parameters of the prepared sample are shown in Table 1, where theoretical mass density (TMD) was used to characterize the density and represented the ratio of actual density to theoretical density. A 14.5 mm ballistic gun was used to carry out ballistic impact experiments, and the experimental device was a quasi-sealed chamber device. The specific experimental procedure was the same as that in the literature [30,31]. The high-speed camera (Phantom VEO 710, VRI Ltd., Wayne, NJ, USA) recorded the impact process of the fragments. The impact energy release of the material can be obtained by combining the fragment velocity and the overpressure value measured by the sensor. The reaction products were recovered and characterized to analyze the energy release characteristics during the impact process. The impact experiments were conducted at room temperature.

## 3. Experimental Results

### 3.1. Microstructure

**Figure 4:**
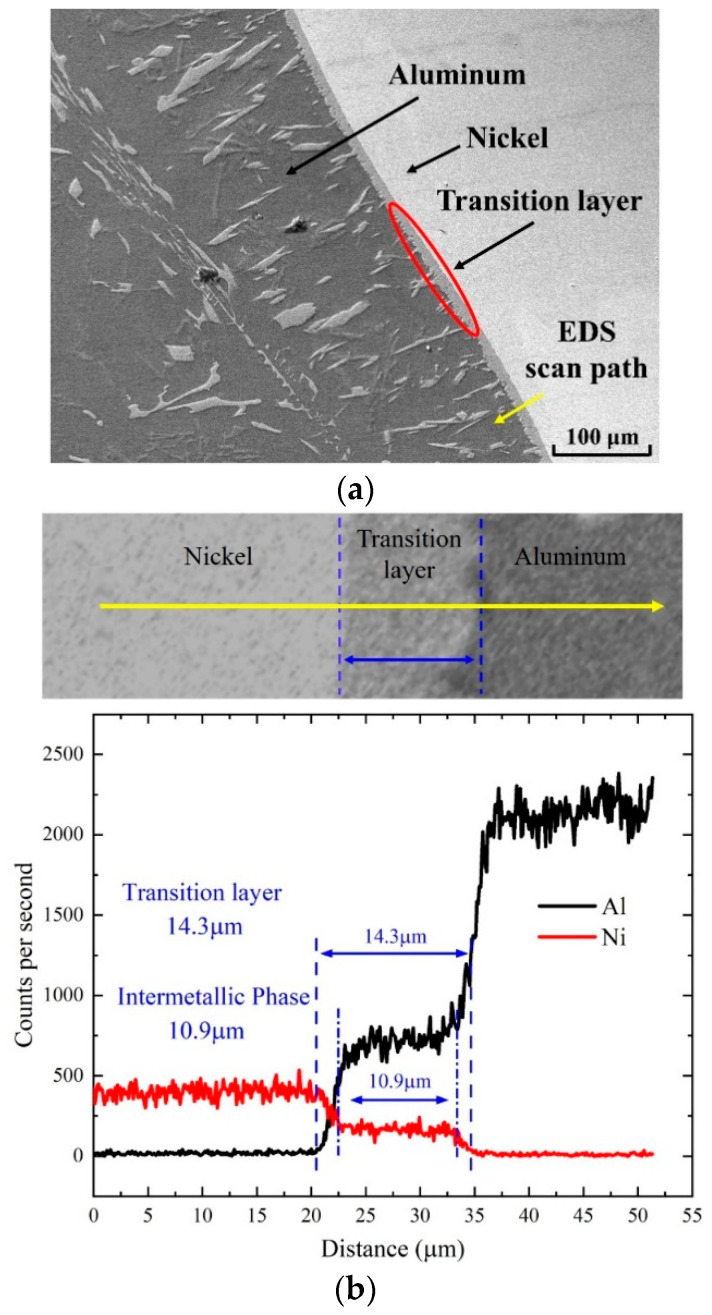
Skeleton-structured Al/Ni reactive material: (**a**) results of SEM test and EDS line scan path; (**b**) results of EDS line scan.

The morphology of the material is shown in Figure 4a. The darker part of the figure is aluminum, and the lighter part is nickel. There is a light gray transition between the two metals. As shown in the figure, the light gray area has a uniform thickness along the metal but there are jagged and dendritic edges near the position of aluminum. It is possible that the transition layer was formed via Ni elements diffusing into the molten Al.

For the position of the transition area, line scanning analysis of aluminum and nickel elements was performed along the direction perpendicular to the interface. The scanning path is indicated by the yellow arrow in Figure 4a, and the line scanning analysis results are shown in Figure 4b. As shown in the figure, three positions of the nickel region, transition region, and aluminum region are, respectively, passed during scanning. When scanning the transition area, it is found that the proportion of aluminum and nickel elements remains unchanged within a certain range, which indicates that there are aluminum–nickel compounds in the transition area, which shows that when molten aluminum is poured into the nickel plate, there will be intermetallic compounds generated at the aluminum–nickel junction. Through the data obtained from the line scanning analysis results, the ratio of aluminum to nickel was calculated. It can be seen that the ratio of aluminum to nickel atoms in the intermetallic compound layer region is about 3:1, which indicates the intermetallic compound is Al_3_Ni.

### 3.2. Test Results of Mechanical Properties

#### 3.2.1. Quasi-Static Compression and Tension Tests

**Figure 5:**
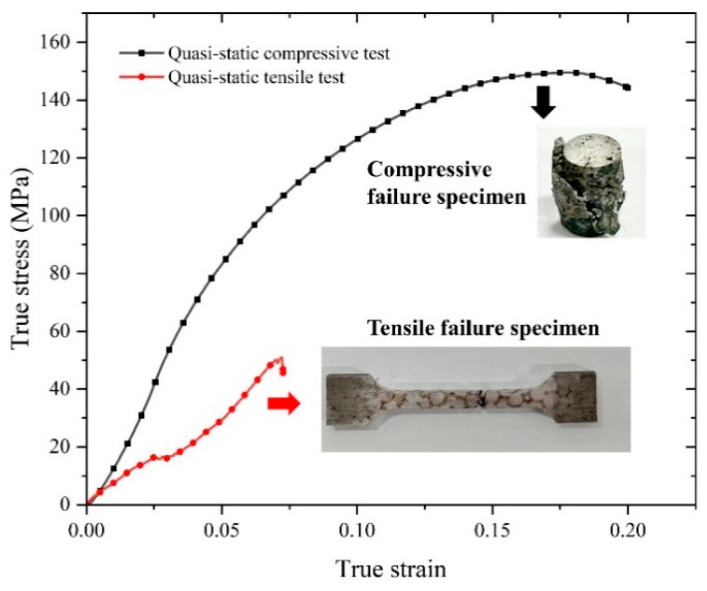
Quasi-static compressive and tensile stress–strain curves of skeleton-structured material and failure specimen.

Figure 5 shows the quasi-static compression and tensile true stress–strain curves of skeleton-structured Al/Ni reactive materials at an initial strain rate of 10^−3^ s^−1^. In the process of compression deformation, it is mainly divided into three stages. The first is the elastic deformation stage of the material, where there is no obvious yield effect; then, the material goes through the obvious plastic deformation stage—with the increase in strain, there is an obvious stress enhancement phenomenon—and then it is the fracture failure stage, where when the strain increases, the stress gradually decreases until the fracture. The specimen has the characteristics of a shear fracture, and the crack expands diagonally at a certain angle. At the same time, it also has the characteristics of a splitting fracture, resulting in axial splitting cracks parallel to the loading direction. The nominal yield stress can reach 68 MPa, the ultimate stress can reach 150 MPa, and the plastic strain can reach 17.8%. It has good plastic deformation capacity. Under quasi-static tensile conditions, the yield stress is up to 16 MPa, the ultimate stress is about 51 MPa, and the failure strain is 7.2%, all of which are significantly lower than the stress and strain under quasi-static compression conditions. The specimen is fractured near the middle area, showing obvious tensile fracture characteristics but no obvious necking phenomenon.

#### 3.2.2. SHPB Test

**Figure 6:**
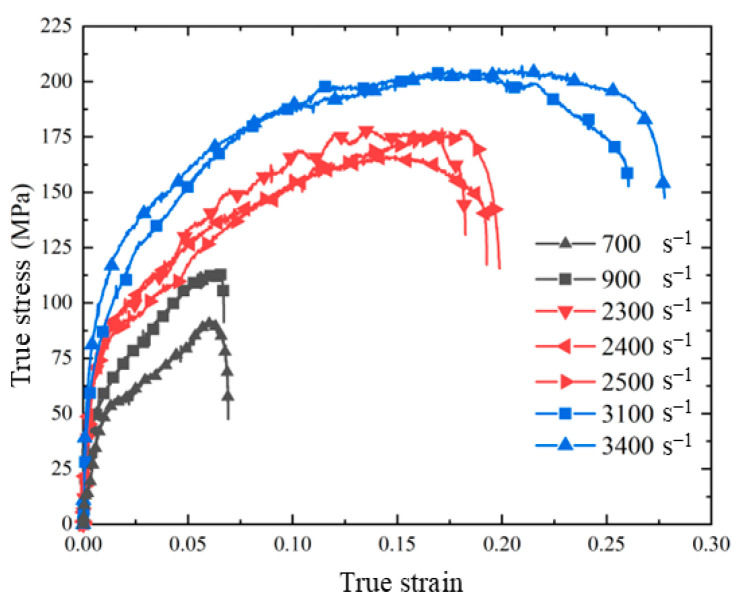
Dynamic compression test results of skeleton-structured Al/Ni reactive material at room temperature.

The collected voltage signal was processed to obtain the dynamic compression test results of skeleton-structured Al/Ni reactive material at room temperature. As shown in Figure 6, under the dynamic load, the real stress–strain curve of the material first shows the linear elastic behavior, followed by the obvious plastic deformation stage. After the material reaches the yield stage, with the increase in plastic strain, the increase in stress is relatively large, and it has obvious work hardening behavior. With the increase in strain rate, both the yield stress and ultimate stress of the material increase significantly, which indicates that the skeleton-structured Al/Ni reactive material has a significant strain rate strengthening effect at high strain rates (700 s^−1^~3400 s^−1^). The yield stress and ultimate stress of the material under various strain rate conditions are shown in Table 2. The ultimate stress of the material increases significantly when the strain rate increases, but the yield stress does not increase significantly after reaching 2300 s^−1^.

### 3.3. Fracture Morphology

#### 3.3.1. Quasi-Static Compression Failure

**Figure 7:**
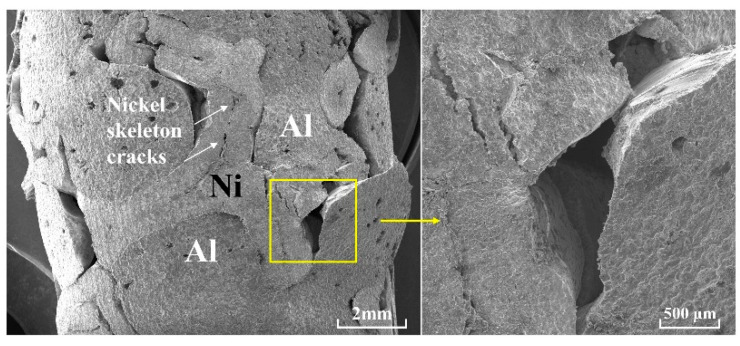
Side morphology of the skeleton structure with Al/Ni reactive material after failure fracture.

Figure 7 shows the side view morphology of skeleton-structured Al/Ni reactive material after quasi-static compression. The picture on the right is the local enlarged picture on the left. After the specimen is compressed, large pieces of material are extruded on the side. Not only is the connection between the aluminum matrix and the nickel skeleton destroyed but also the nickel skeleton and the aluminum matrix themselves are broken. The bonding surface between aluminum and nickel has holes and voids; the specimen will first expand outward after compression, and the defect at the bonding surface will appear as stress concentration. With the increase in stress, the holes and voids will gradually expand to form cracks, and cracks will extend along the bonding surface. Under the action of shear force, when the crack grows to a certain extent, the bonding surface with more defects will be disbanded as a whole, and the bonding surface will show a smooth shear slip pattern, as shown in the right figure. The fracture section at the lower left of the fracture section does not show shear slip but an irregular pattern formed by tearing and pulling. According to the deformation form of the fracture in the left figure, when no failure occurs here, the interface of the aluminum matrix and nickel metal skeleton has a certain angle to the axial direction. When the pressure gradually increases, the section will shear slip due to the change in the force direction caused by the angle, and finally, the entire aluminum matrix will be removed, and other parts will be pulled along the interface. After tearing, irregular lines are formed. When the bonding surface is damaged, the nickel skeleton and aluminum matrix will also be affected by the stress concentration; cracks will appear at the stress concentration, and crack propagation will also lead to the destruction of the nickel skeleton and aluminum matrix. On the nickel skeleton, there are many cracks along the inherent defect stress concentration, and with the crack expansion, the nickel skeleton will fracture.

#### 3.3.2. Quasi-Static Tensile Failure

**Figure 8:**
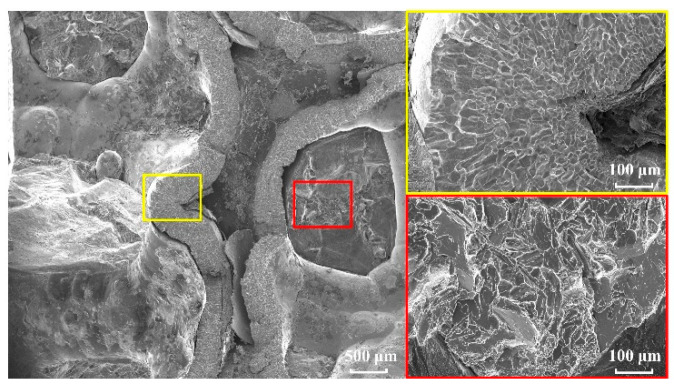
Microstructure of fracture section of the material at a 10^−3^ s^−1^ strain rate.

Figure 8 shows the microstructure of the fracture cross-section of the skeleton-structured Al/Ni reactive material under quasi-static tensile conditions at a 10^−3^ s^−1^ strain rate. The picture on the right shows enlarged images of the yellow and red regions, corresponding to the micromorphologies of fracture cross-sections of the nickel skeleton and aluminum matrix. Regarding the aluminum matrix in the left part of the yellow region, because its fracture section is not perpendicular to the direction of stress, the scratch of shear failure can be seen on it, and its fracture form is different from that of the aluminum matrix in the red region. The fracture section in the yellow region on the right shows a bright reflective surface, which indicates that the nickel skeleton has an intergranular fracture under the action of tensile load. The red region fracture section has an obvious step-like pattern and gloss surface with high reflectivity. There is a large crack due to the preparation of the casting process, so the solidification of aluminum after the completion of the pouring will inevitably produce bubbles; aluminum itself may be the cause of large cracks. However, different from the microscopic morphology of the cleavage fracture, this fracture section is obviously rougher and appears in the form of pulling and tearing, and only smooth fracture sections can be found locally. Judging from the difference in fracture morphology, the fracture form of the aluminum matrix should be a quasi-cleavage fracture, which is located in a transition state between a ductile fracture and cleavage fracture [32].

#### 3.3.3. Dynamic Compression Failure

**Figure 9:**
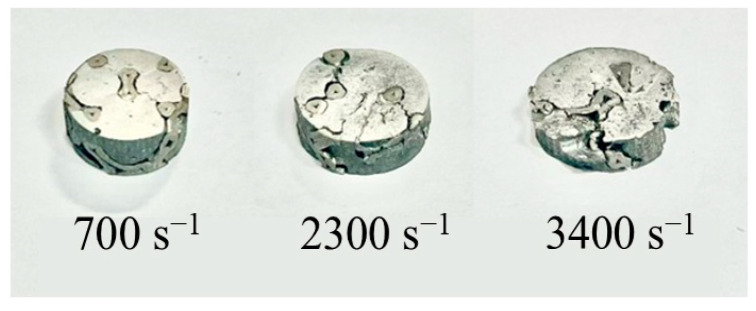
Dynamic compressive deformation of the sample at different strain rates.

As shown in Figure 9, under dynamic loading, the sample was upset by the impact force. Different from the quasi-static compression test, under a high strain rate, the specimen will become flat, and the separation between the skeleton and the matrix can be found at the end face. With the increase in strain rate, the damage degree of the skeleton and matrix will gradually increase, and the crack expansion will gradually break the specimen into small pieces.

**Figure 10:**
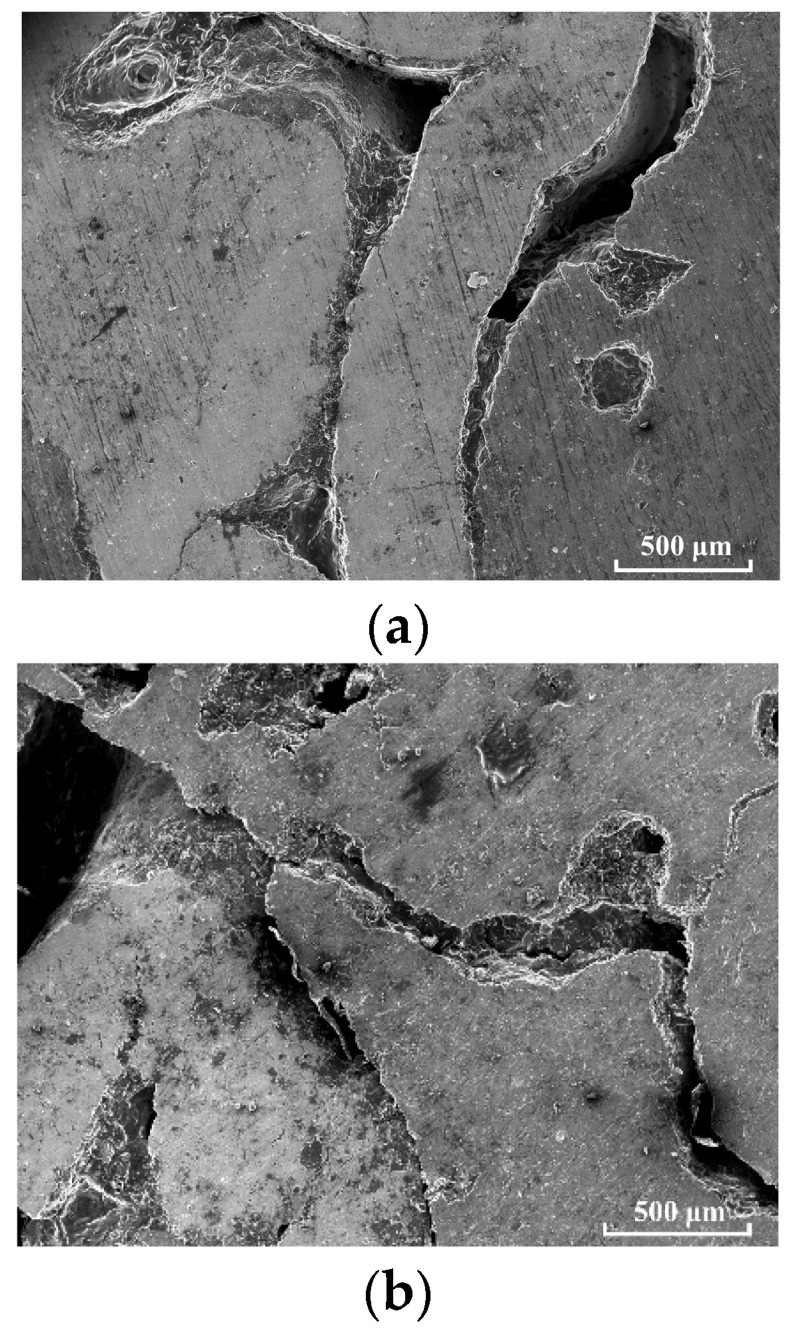

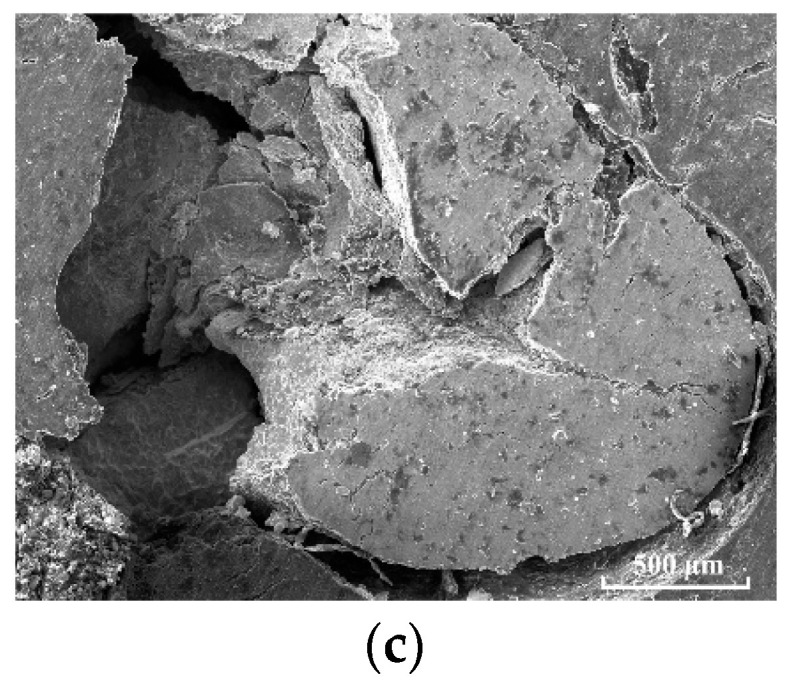
Micromorphologies of specimens at different strain rates: (**a**) 700 s^−1^; (**b**) 2300 s^−1^; and (**c**) 3400 s^−1^.

Figure 10 shows the micromorphologies of the specimens under different strain rates. Figure 10a shows the microstructure of the specimen at the strain rate of 700 s^−1^. After being impacted, the aluminum–nickel bonding surface of the specimen will debond, and the failure mode of pulling and tearing will appear at the interface. The damaged area is still partially connected, and no shear slip form is found. After the specimen is pressed, the radial tensile stress leads to damage on the end face. There will be stress concentration at the inherent defects of the nickel skeleton, resulting in the formation and propagation of cracks along the sharp corners of the defects. In the stress concentration part at the aluminum–nickel interface, the nickel skeleton will also have cracks. Figure 10b shows the microstructure of the sample at the strain rate of 2300 s^−1^. The nickel skeleton has significantly more cracks at the inherent defects, and the degree of crack propagation is greater. The nickel skeleton has an intergranular fracture, and tooth lines are formed at the fracture. The cracks on the aluminum matrix are also larger than those on the low strain rate condition, and there are more holes in the matrix. Figure 10c shows the microstructure of the sample at the strain rate of 3400 s^−1^. When the strain rate continues to increase, the cracks on the nickel skeleton at the end face of the sample will continue to expand, resulting in the intergranular fracture failure of a part of the nickel skeleton, and the fracture surface presents a toothed grain. The interface between the skeleton and the matrix is completely unstuck, and the interface also presents irregular lines formed by pulling and tearing. There are also more holes and cracks in the aluminum matrix.

### 3.4. Quasi-Sealed Chamber Experiment Results

**Figure 11:**
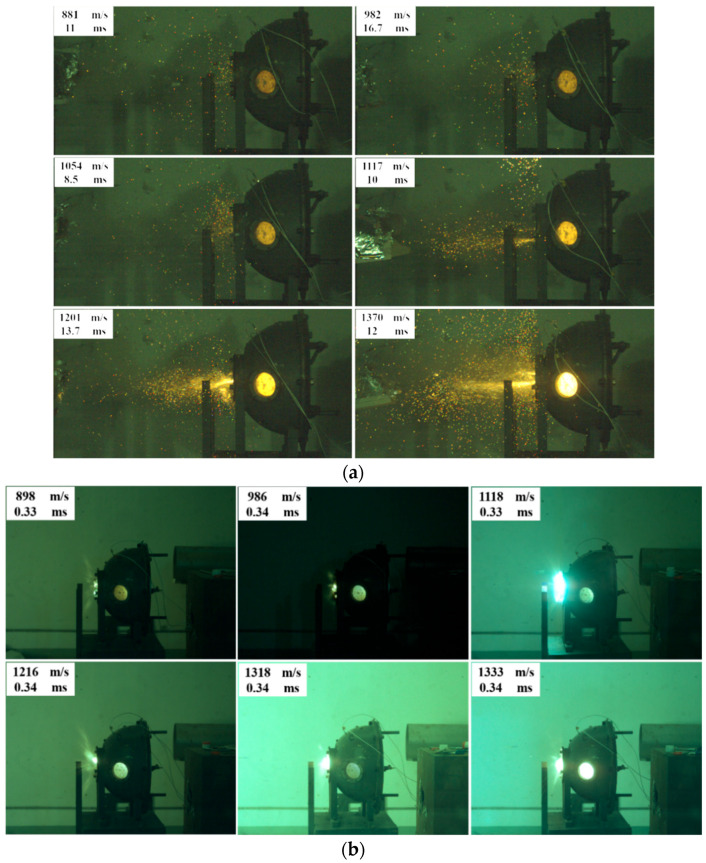
High-speed video screenshots of the impact reaction of skeleton-structured Al/Ni reactive materials at different velocities: (**a**) under the atmosphere of air and (**b**) under the atmosphere of argon.

To study the impact velocity and gas atmosphere inside the chamber on the impact reaction process of skeleton-structured Al/Ni reactive materials, impact experiments were conducted under different gas atmospheres. The impact energy release characteristics are shown in Figure 11, which marks the data of the brightest moment of impact velocity and spark ejection phenomenon. It can be seen from Figure 11a that in the air atmosphere, the sample has an obvious spark ejection phenomenon after impact, which proves that the material has a violent chemical reaction inside the chamber. With the increase in the impact velocity, the spark ejection phenomenon becomes more and more obvious, and the brightness of the observation window is also significantly enhanced, which indicates that the impact velocity can significantly affect the reaction of the reactive material in the chamber, making the reaction more intense.

As shown in Figure 11b, there is almost no spark ejection phenomenon of the material under the impact condition in the argon atmosphere. Even if the impact velocity of the reactive fragment increases, there is no spark ejection phenomenon, and the results of high-speed video are nearly the same at all impact velocities. This shows that the material does not have a strong energy release phenomenon in the argon atmosphere; the reaction of the material under the impact condition may need the support of oxygen. Under the atmosphere of argon, increasing the impact velocity of the fragment can not improve the reaction degree of the material, and it can be preliminarily judged that there may not be a violent intermetallic combination reaction between the Al and Ni or a very low degree of reaction.

**Figure 12:**
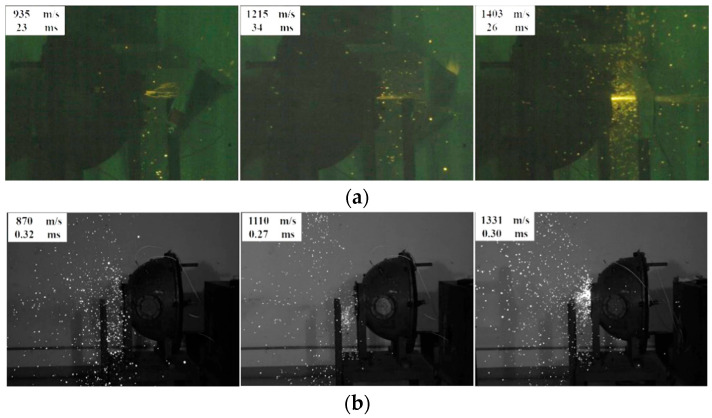
High-speed video screenshots of impact energy release of cold-pressed Al/Ni reactive materials at different velocities: (**a**) under the atmosphere of air and (**b**) under the atmosphere of argon.

Figure 12 shows the high-speed video screenshots of cold-pressed Al/Ni reactive materials under different gas atmospheres. As shown in Figure 12a, under the atmosphere of air, the cold-pressed Al/Ni reactive material has a spark injection phenomenon, and the greater the impact velocity, the more obvious the spark injection phenomenon. As shown in Figure 12b, under the atmosphere of argon, the cold-pressed material and the skeleton-structured material produce a similar phenomenon, and no spark injection phenomenon is found in either of them, and there is no obvious bright light in the observation window. By comparing the energy release characteristics of the two materials under the atmosphere of air, it can be found that at the same velocity, the spark injection phenomenon of the cold-pressed Al/Ni reactive material is weaker than that of the skeleton-structured Al-Ni reactive material, and the skeleton-structured Al/Ni reactive material has a stronger impact energy release ability. Combined with the above analysis of the energy release phenomenon of different reactive materials under different gas atmospheres, it can be considered that the impact energy release of cold-pressed Al/Ni reactive materials also needs the support of oxygen in the air, and the intermetallic combination reaction between the components of the material is difficult to carry out.

**Figure 13:**
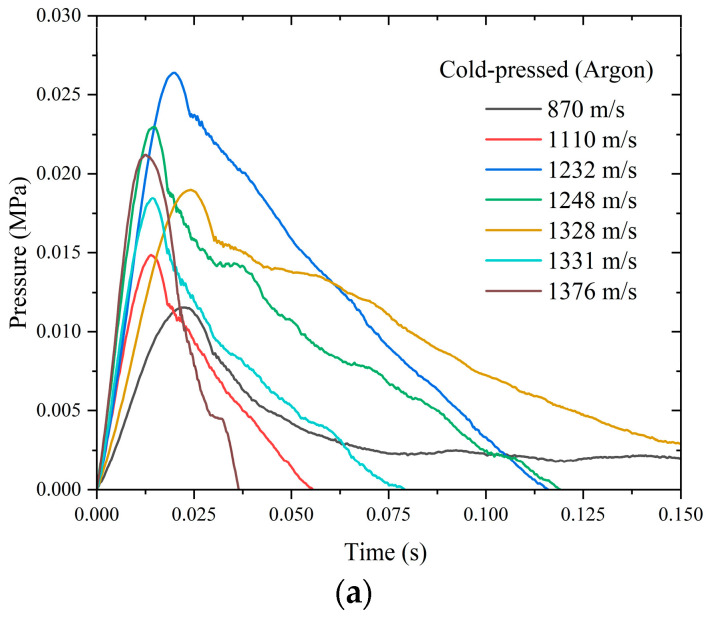

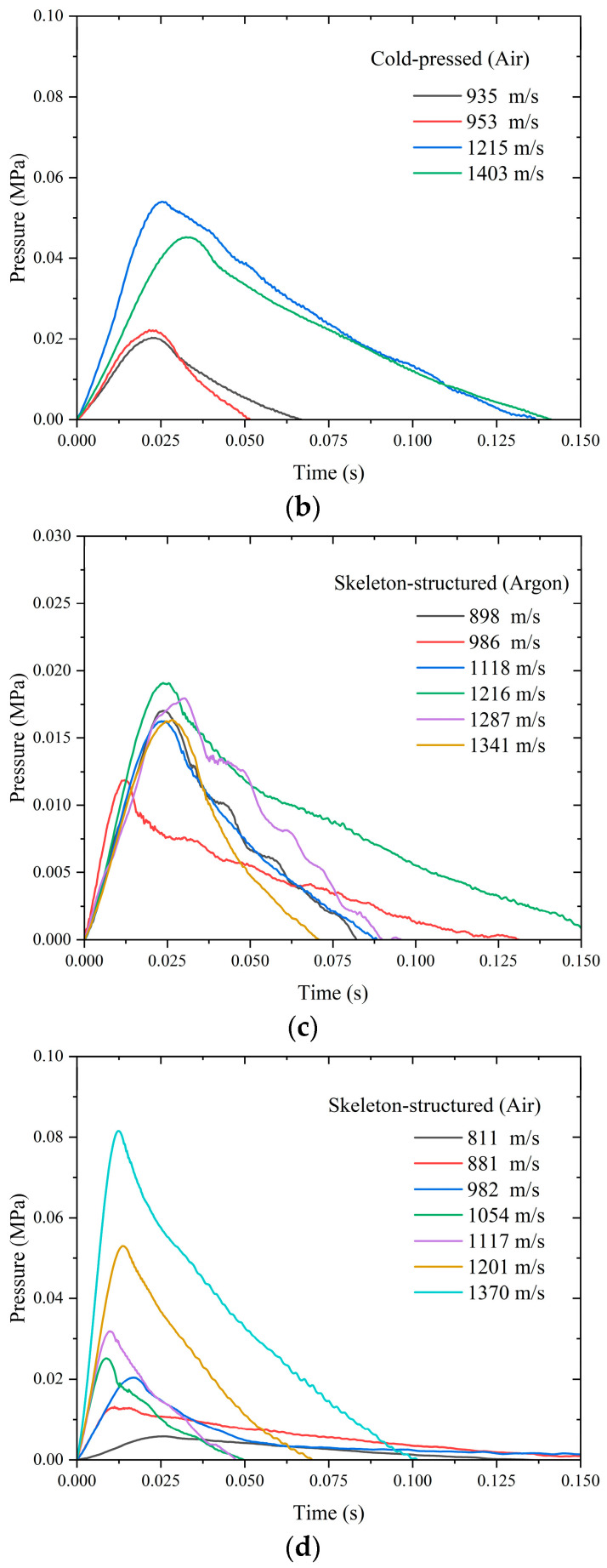
Quasi-static pressure and time curves of the two materials under the different gas atmospheres: (**a**) cold-pressed material under the atmosphere of argon; (**b**) cold-pressed material under the atmosphere of air; (**c**) skeleton-structured material under the atmosphere of argon; and (**d**) skeleton-structured material under the atmosphere of air.

Figure 13 shows the quasi-static pressure and time curves of the treated skeleton-structured Al/Ni reactive material and the cold-pressed Al/Ni reactive material under different gas atmospheres and different impact velocities. Where (a) and (b) are the test results of the cold-pressed Al/Ni reactive material under the atmosphere of argon and air, respectively, (c) and (d) are the test results of the skeleton-structured Al/Ni reactive materials under the atmosphere of argon and air, respectively.

As shown in Figure 13a, under the atmosphere of argon, the quasi-static pressure performance of cold-pressed Al/Ni reactive materials under different impact velocities is almost the same, and the quasi-static pressure peak value is the same. When the velocities are 870 m/s~1376 m/s, the quasi-static pressure peak value is within the range of 0.01 MPa~0.025 MPa. It can be seen from Figure 13c that the quasi-static pressure performance of skeleton-structured Al/Ni reactive materials under the atmosphere of argon is similar to that of cold-pressed Al/Ni reactive materials. At the range of 898 m/s~1341 m/s, the quasi-static pressure peak value of skeleton-structured Al/Ni reactive materials is in the range of 0.015 MPa~0.02 MPa. Its peak value distribution is denser than that of cold-pressed reactive materials, and the performance is almost identical at the impact velocities of 898 m/s, 1118 m/s, 1287 m/s, and 1341 m/s. The results of quasi-static pressure data under the atmosphere of argon show that, for the two different preparation processes of Al/Ni reactive materials, the increase in impact velocity cannot increase the quasi-static pressure, and the impact velocity has little correlation with the quasi-static pressure.

It can be seen from Figure 13b that under the air atmosphere, the peak value of the quasi-static pressure of the cold-pressed Al/Ni reactive material gradually increases with the increase in impact velocity, and the peak time also gradually moves forward with the increase in impact velocity. The peak pressure of the material at 1215 m/s and 1403 m/s is about twice as large as that at 935 m/s and 953 m/s and is also larger than that under the atmosphere of argon. It can be seen from Figure 13d that under the atmosphere of air, the quasi-static pressure peak of skeleton-structured Al/Ni reactive materials is also positively correlated with the impact velocity. The impact velocity can significantly increase the quasi-static pressure peak value of the materials, while the peak pressure time will also move forward, and the quasi-static pressure peak value will increase by about 1.5 to 2 times every time the velocity increases by 100 m/s. The quasi-static pressure curve gradually becomes steeper with the increase in impact velocity, and the slope of the curve gradually increases, which indicates that, in this case, the reaction efficiency of the material is improved, and so is the pressure performance of the cold-pressed Al/Ni reactive material. The increase in impact velocity enhances the reaction efficiency of the reactive material. The peak pressure data of the skeleton-structured Al/Ni reactive material at 1370 m/s are about 0.03 MPa more than that of the cold-pressed Al/Ni reactive material at a similar velocity. At a higher impact velocity, the impact energy release of the skeleton-structured Al/Ni reactive material is better. In comparison, the quasi-static pressure peaks of the two materials are almost the same under the atmosphere of argon, but under the atmosphere of air, the reaction of the skeleton-structured Al/Ni reactive materials is significantly more intense.

**Figure 14:**
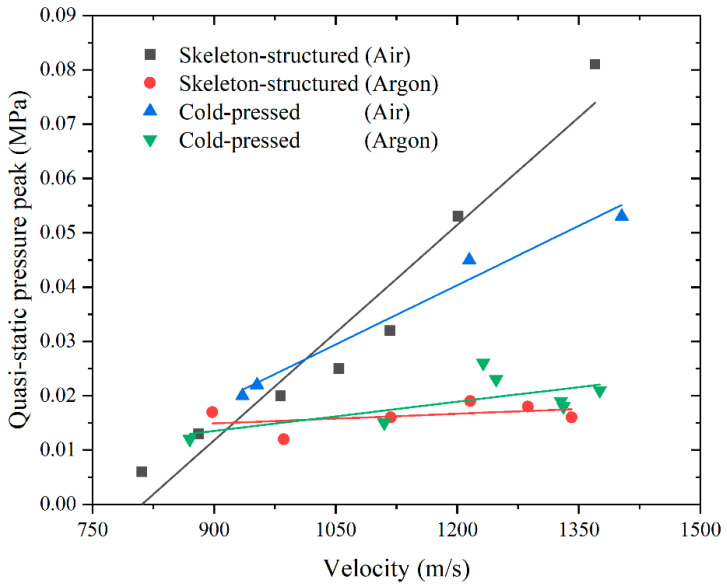
Relation of quasi-static pressure peak to impact velocity under different atmospheres and materials.

Table 3 and Figure 14 show the relationship between the peak value of quasi-static pressure and the impact velocity under different gas atmospheres and materials. It can be found that under different atmospheres, the peak value of pressure inside the chamber will increase with the increase in the impact velocity. When the impact velocity is relatively small (800 m/s~900 m/s), the overpressure peaks of the two materials under the two gas atmospheres are relatively close. When the impact velocity is large (900 m/s~1400 m/s), the overpressure peaks of the two materials in the argon atmosphere are consistent. When the impact velocity is in the range of 800 m/s~1200 m/s, the overpressure peak value of the two materials under the atmosphere of air is slightly different, but after the velocity exceeds 1200 m/s, the pressure peak value of the skeleton-structured Al/Ni reactive material is significantly higher. This shows that under the atmosphere of argon, the impact velocity has a relatively small influence on the quasi-static pressure. Under the atmosphere of air, the impact velocity on the static pressure is more obvious, and the impact pressure gain effect of the skeleton-structured Al/Ni reactive material is greater than that of the cold-pressed Al/Ni reactive material. It can be seen from the analysis results of peak pressure that the reaction strength of the skeleton-structured Al/Ni reactive material is higher than that of the cold-pressed Al/Ni reactive material, and the reaction is more sensitive to the impact velocity and more intense. Based on the above test results, it can be concluded that the main reason for the inconsistent impact reaction results of skeleton-structured Al/Ni reactive materials under different atmospheres is that the main chemical reaction of the material under such conditions is the oxidation reaction of aluminum and nickel rather than the intermetallic combination reaction, and the cold-pressed Al/Ni reactive materials also have such characteristics of an easy oxidation reaction.

## 4. Numerical Simulation of Mechanical Properties

### 4.1. Material Model

**Figure 15:**
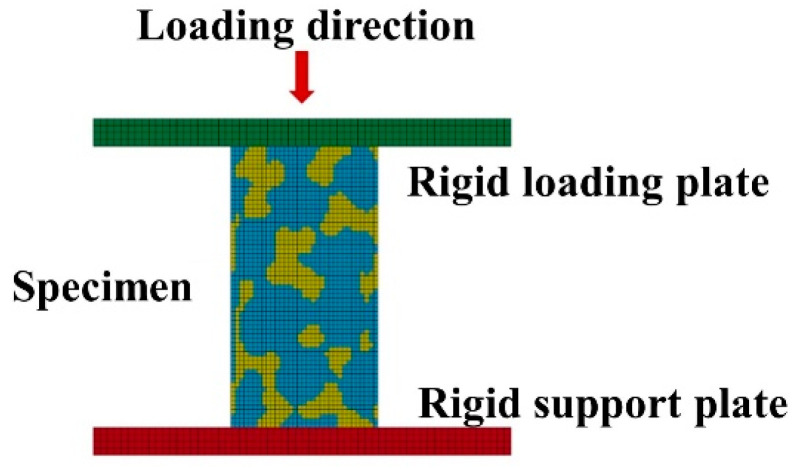
Quasi-static compression numerical simulation model.

Combined with Mimics medical image analysis software, the mesh mapping method [33,34] was used to comprehensively consider the influence of the mesh type and model size on the simulation, and combined with the CT scan results of the nickel skeleton structure, the reconstruction of a nickel skeleton and aluminum filler in the skeleton-structured Al/Ni reactive material was carried out, and the grid hexahedral mapping grid was divided. A cylinder with a size of Φ10 mm × H20 mm was cut out from the established composite material model, in which the yellow grid represents the nickel skeleton and the blue grid represents the aluminum filler, as shown in Figure 15. Quasi-static compression calculations were carried out using LS-DYNA commercial software (ANSYS, Canonsburg, PA, USA), and the form of a rigid loading plate and support plate simulated the indenting head and base of the testing machine. The rigid loading plate was set to load downward at a constant rate of 0.02 mm/s, and the compressive strain rate was 10^−3^ s^−1^.

**Figure 16:**
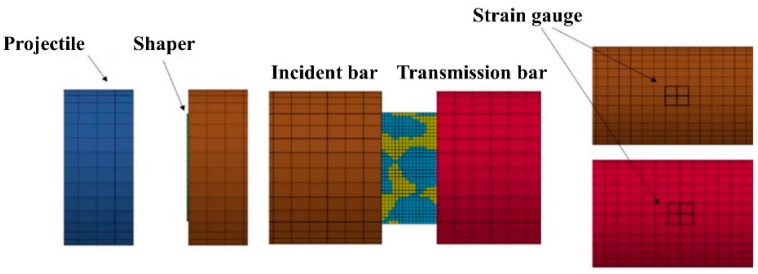
Dynamic compression numerical simulation model.

In the numerical simulation of dynamic compression of the skeleton-structured Al/Ni reactive materials, the finite element mesh model adopted was consistent with the quasi-static compression simulation model, and the model size was changed to a flat cylinder of Φ10 mm × H5 mm, as shown in Figure 16, which was consistent with the sample size of the dynamic mechanical properties test.

### 4.2. Simulation Model

In the computational model of the numerical simulation for quasi-static compression, the rigid plate material adopts the MAT_RIGID model, which can simulate the compression situation under quasi-static tests well. In numerical simulation, a rigid loading plate is used to replace the indenter of the testing machine. Both aluminum and nickel materials adopted the MAT_024(MAT_PIECEWISE_LINEAR_PLASTICITY) model. This model is a multi-segment elastoplastic material model that can describe the nonlinear stress–strain relationship and requires the input of true stress–strain curve data under uniaxial conditions for the plastic part of the material. The material parameters of aluminum and nickel in this chapter all take into account the effect of failure on the simulation. The added failure parameters are determined by the measured failure strain. In the numerical simulation of dynamic compression, the bar and shaper are defined as variable bodies, and the specific material parameters of the bar and shaper are determined according to reference [35]. To consider the consistency of the material parameters, the MAT_098(MAT_SIMPLIFIED_JOHNSON_COOK) model, whose material parameters are consistent with those in the quasi-static compression numerical simulation, is adopted in this paper. This model is a simplified JC model, which does not consider the temperature rise in the material at a high strain rate and can significantly improve the computational efficiency.

#### 4.2.1. Quasi-Static Simulation

**Figure 17:**
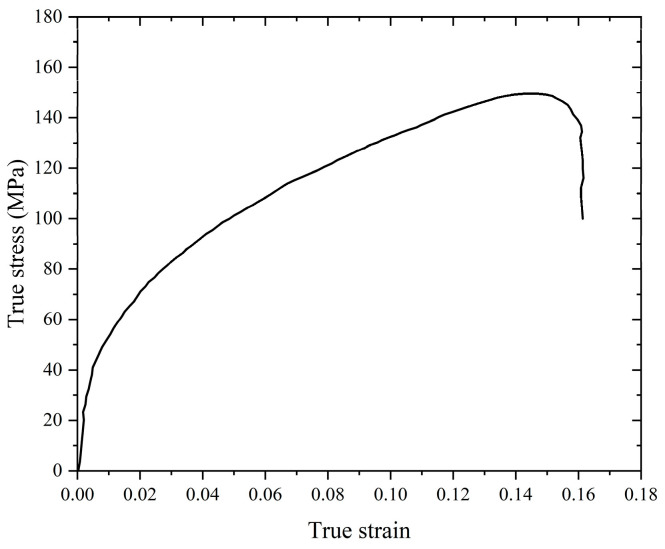
Real stress–strain curve relationship of quasi-static compression numerical simulation.

Figure 17 shows the real stress–strain curve relationship of the quasi-static compression numerical simulation for skeleton-structured Al/Ni reactive materials. Based on the analysis of the curve, it can be seen that the simulation model roughly goes through the following three stages: elastic deformation stage, plastic deformation stage, and fracture failure stage. The simulation results are compared with the test results, and the results are shown in Table 4. It can be seen that in the simulation results, the yield strain and plastic strain of the material are lower, and the yield stress is also lower, while the ultimate stress is close to the actual test results. The material quickly reached the yield stage in the compression process, probably because the co-node method could not fully fit the metallurgical connection between the aluminum–nickel phase and the defects and holes between the aluminum–nickel bond surface. The intermetallic compound between the aluminum–nickel phase and the connection between the two phases made the mechanical response of the material in the elastic stage different from the numerical simulation. The effect of this compound layer on the material is difficult to achieve in numerical simulations.

Due to the preparation process, the nickel matrix is filled with molten aluminum. The aluminum solidified after melting will form new grains when re-curing, affecting the distribution of impurities and defects in the material, so the parameters of the material model may not be completely consistent with the actual material. In addition, this model is a cylinder model obtained by random excision from CT modeling. The integrity of the nickel skeleton in this model and the proportion and density of filled aluminum will also affect the final result. Although the simulation results reflect some problems that cannot completely fit real situations of the material, the stress–strain curve results obtained by the numerical simulation and the results obtained by the real test are not much different in value; the ultimate stress is very close but the performance is inconsistent in the deformation form, which also shows that the established model can correctly reflect the real results to a certain extent. It can provide a basis for the subsequent numerical simulation research and a real test of skeleton-structured Al/Ni reactive materials.

**Figure 18:**
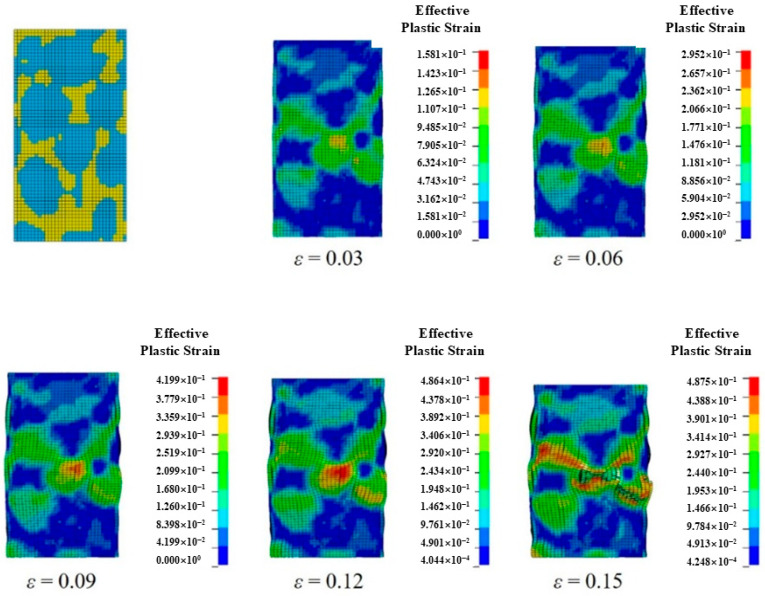
Quasi-static compressive equivalent plastic strain distribution of skeleton-structured Al/Ni reactive materials.

Figure 18 shows the distribution of equivalent plastic strains of the finite element model under different strains. With the increase in strain, the material produces a large plastic deformation, which leads to the failure and fracture of the model when it exceeds the set threshold. During the gradual increase in strain from 0 to 0.15, the model goes through elastic deformation and plastic deformation stages and will gradually expand outward. In the process of expansion, the forces of each part are not uniform, resulting in stress concentration. The equivalent plastic strain of aluminum is greater than that of nickel. It can be found that the increase in equivalent plastic strain mainly occurs in the direction of a 45-degree angle with the axial direction of the model, the plastic deformation is mainly concentrated in the shear zone, and the stress in the plastic deformation stage rises rapidly, finally leading to the shear fracture of the model. When a shear fracture occurs, in the direction of 45 degrees, part of aluminum has fracture failure; nickel has not failed, however, where only a more obvious bending deformation has occurred.

#### 4.2.2. Numerical Simulation of Dynamic Compression

**Figure 19:**
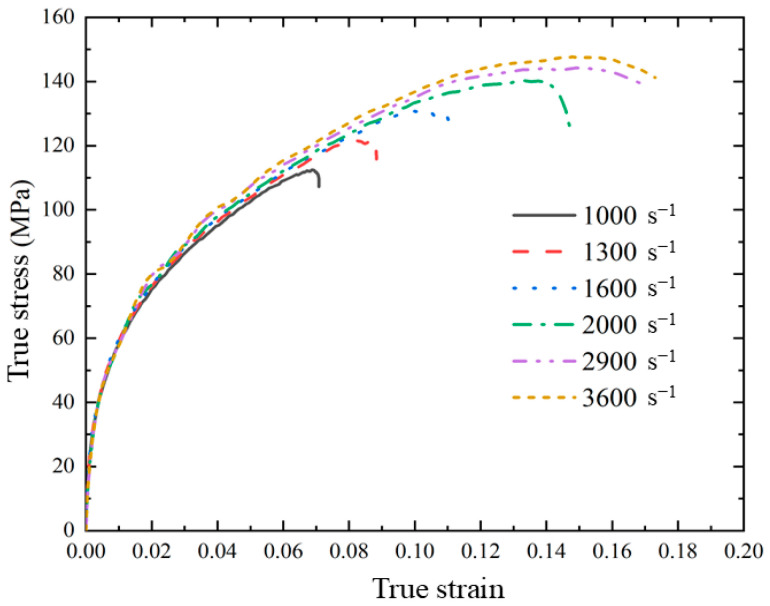
Real stress–strain curves of dynamic compression numerical simulation.

After processing the obtained strain data, the real stress–strain curves of the dynamic compression numerical simulation are shown in Figure 19. It can be found that under the conditions of different strain rates, all curves are consistent in the yield stage and different only under the ultimate stress and ultimate strain. The real stress–strain curves first show the linear elastic behavior, followed by the plastic deformation stage. The material shows obvious work hardening behavior in the numerical simulation. With the increase in the strain rate, the flow stress and ultimate stress increase. When the numerical simulation strain rate is 1000 s^−1^, the real stress–strain curve can correspond well with the stress–strain curve under the condition of the real test at the strain rate of 900 s^−1^, but under the condition of a higher strain rate, the numerical simulation can not match the real situation well, and the ultimate stress of the numerical simulation is low, and the influence of the strain rate is small. The ultimate strain results are in good agreement with the real ones. The data of the numerical simulation under different strain rates are shown in Table 5.

It can also be found in the table that under different strain rates, the yield stress results of the numerical simulation do not significantly enhance or change. Compared with the real test results of SHPB, the yield stress of the skeleton-structured Al/Ni reactive materials at different strain rates is also consistent under real conditions, but the ultimate stress increases significantly with the increase in the strain rate. It can be considered that the special structure inside the material leads to the fact that its yield stress does not increase with the increase in the strain rate. Moreover, since the intermetallic compound layer is contained between the two phases of aluminum–nickel, the common node grid model cannot predict the yield stress of the material well in the numerical simulation, resulting in no significant change in the yield stress under each strain rate condition in the numerical simulation results. There is also a certain gap between the numerical simulation results and the actual test results. In the numerical simulation, the increase in strain rate does not significantly increase the ultimate stress. In the real test, the increase in strain rate will significantly improve the ultimate stress of the material, and the true stress–strain curves under different strain rates are different. The strain rate hardening effect of the material is obvious, which may be due to the inconsistency between the combination of aluminum and nickel phases and their respective deformation under impact in the real situation. Under dynamic compression conditions, the failure forms of the aluminum matrix and the nickel skeleton are inconsistent. The nickel skeleton appears in the form of an intergranular fracture, while the aluminum matrix is in the form of a shear slip and tensile tear. In the numerical simulation, the mesh is deformed after being subjected to pressure, and the mesh is deleted and fails after reaching a certain extent. In addition, the material model may not be able to accurately predict the mechanical response of the material under the condition of a high strain rate, so the stress–strain curve of the numerical simulation is lower than that of the real result under the condition of a high strain rate.

## 5. Discussion

### 5.1. Calculation of Impact-Induced Energy Release Efficiency

To use quasi-static pressure changes to calculate the chemical energy released by the reactive material, it is necessary to align the air inside the chamber to make assumptions. First, within the time range when the quasi-static pressure reaches the highest point from zero, it is believed that the part of the air inside the chamber that escapes through the bullet hole at the front of the chamber does not affect the overall calculation result. Second, at this time range, only a small number of projectiles participate in the gas phase during the impact reaction process, and the air density inside the chamber does not change. Since the density does not change, while considering the above assumptions, it is considered that the changes in the internal mass of the chamber and the internal pressure of the constant volume chamber can be calculated using the following formula:

(1) $$\Delta P=R\rho \left(T_{2}-T_{1}\right)$$

where *R* is the gas constant of air; for an ideal gas, this is the difference between a specific heat capacity at a constant pressure and a specific heat capacity at a constant volume, which can be regarded as a constant value; *ρ* is the density of the gas inside the chamber; and *T*_1_ and *T*_2_ are the temperatures of the air under these two states, respectively. Assuming that the internal volume of the quasi-sealed chamber is *V*, the increment of gas energy can be expressed as:

(2) $$\Delta E=\rho VC_{V}\left(T_{2}-T_{1}\right)$$

where Δ*E* is the increment of the gas energy inside the chamber, and *C*_V_ is the specific heat capacity of the gas at a constant volume. The specific heat capacity at a constant volume can be calculated by the following formula:

(3) $$C_{V}=\frac{R}{\gamma -1}$$

where *γ* is the specific heat capacity ratio of a gas, which is usually 1.4 for air. Substitute Formula (2) and (3) into (1) to obtain:

(4) $$\Delta E=\frac{V}{\gamma -1}\Delta P$$

When the reactive fragment passes through the thin lid at the front of the quasi-sealed chamber, the velocity will decrease to a certain extent. To simplify the calculation of kinetic energy, relevant scholars assume that the kinetic energy lost by the reactive fragment when it penetrates the thin lid at the front of the chamber accounts for 10% of the total kinetic energy. The remaining kinetic energy of the fragments when they impact the hardened steel target in the quasi-sealed chamber is 90% of the initial kinetic energy [24], or the impact of the front skin of the chamber on the fragment velocity is not considered. The THOR equation was used to calculate the residual velocity of reactive fragments after passing through the quasi-sealed chamber [36], and the formula was shown as follows:

(5) $$V_{r}=V-0.3048\times 10^{c_{1}}{\left(61023.75HA^{’}\right)}^{c_{2}}{\left(15432.1m\right)}^{c_{3}}{\left(3.28084V\right)}^{c_{4}}$$

where *V*_r_ is the residual velocity; *H* is the thickness of the lid; *A*^’^ is the collision area between the fragment and the skin; *m* is the mass of the fragment; *c*_1_~*c*_4_ is the skin material parameters, which are 6.399, 0.889, −0.945, and 0.019, respectively. Assuming that the reactive fragment vertically impacts the thin lid and carries the thin lid with the same collision area into the quasi-sealed chamber, without considering the mass loss, the residual kinetic energy of the fragment [36] can be calculated as:

(6) $$E_{k}=\frac{1}{2}\left(m+m_{r}\right)V_{r}^{2}$$

where *m*_r_ is the mass of the lid with the same collision area. The kinetic energy of the fragments will affect the measured quasi-static pressure value to a certain extent, so after calculating the total energy released by the reactive material using the quasi-static pressure value, the chemical energy released by the fragments can be obtained only after the kinetic energy of the fragments is fed back to the partial removal of the quasi-static pressure. According to the quasi-static pressure test results of the steel specimen [30,37], only about 25% of the final kinetic energy of the specimen was converted into the internal energy of the gas, and the heat loss should be ignored in the calculation. According to the test results of the impact energy release characteristics of skeleton-structured Al/Ni reactive materials under different gas atmospheres, the main chemical reaction of the materials under impact conditions is the oxidation reaction of aluminum and nickel. To calculate the impact energy release efficiency of the material, it is necessary to determine the heat released by the reaction of each material through the chemical reaction formula, and it is necessary to calculate the chemical energy of the reaction according to the proportion of aluminum and nickel in the sample. The calculated energy difference in the table above is the chemical energy released by the skeleton-structured Al/Ni reactive material. Through the calculation of the energy difference and the proportion of the reaction chemical energy of the material, the impact energy release efficiency of the material can be obtained. The calculation method is as follows:

(7) $$y=\frac{\Delta E-E_{k}^{’}}{E}$$

where *y* is the energy release efficiency, the degree of the chemical reaction; *E*^’^_k_ is the kinetic energy of the sample fed back to the quasi-static pressure part, 25% of the remaining kinetic energy; *E* is the chemical energy released by the complete reaction of the sample, which is usually calculated directly from the heat release of the chemical reaction occurring in the material. The chemical equation of the oxidation reaction of aluminum and nickel and the heat release of the reaction is as follows:

(8) $$\mathrm{Al}+\frac{3}{2}O_{2}=\frac{1}{2}{\mathrm{Al}}_{2}O_{3}\;\Delta Q=-31,068.5\;J/g$$

(9) $$\mathrm{Ni}+\frac{1}{2}O_{2}=\mathrm{NiO}\;\Delta Q=-4085.95\;J/g$$

According to the above chemical reaction equations and reaction enthalpy, the reaction heat of the complete reaction of skeleton-structured Al/Ni reactive materials can be calculated, as shown in Table 6.

After obtaining the reaction heat of the material, the energy release efficiency of the skeleton-structured Al/Ni reactive material at different impact velocities can be calculated, as shown in Table 7. It can be found that the energy release efficiency of the sample at different velocities is different. With the increase in the impact velocity, the energy release efficiency has a relatively large increase, but the highest energy release efficiency is only 8%. Compared with the results of the impact experiments, it can be found that, although the oxidation reaction degree of the skeleton-structured Al/Ni reactive material under such impact conditions is low, there is still a very obvious spark jet phenomenon, and the peak pressure inside the chamber can reach 0.08 MPa. In the research on hot-pressed Al/Ni reactive materials [38], it was found that when the impact velocity exceeded a certain value, the quasi-static pressure value was similar. In the data processing of allocative static pressure peak in this paper, it is also found that, in the impact experiment of skeleton-structured Al/Ni reactive materials, the measured peak value of quasi-static pressure is also close in some tests, with a velocity greater than 1370 m/s, which indicates that there is a limit value for the reaction degree of Al/Ni reactive materials themselves under such impact conditions. Although the increase in the impact velocity can improve the reaction degree of the reactive material, it will not increase it without limitation.

### 5.2. Impact Energy Release Mechanism Analysis

**Figure 20:**
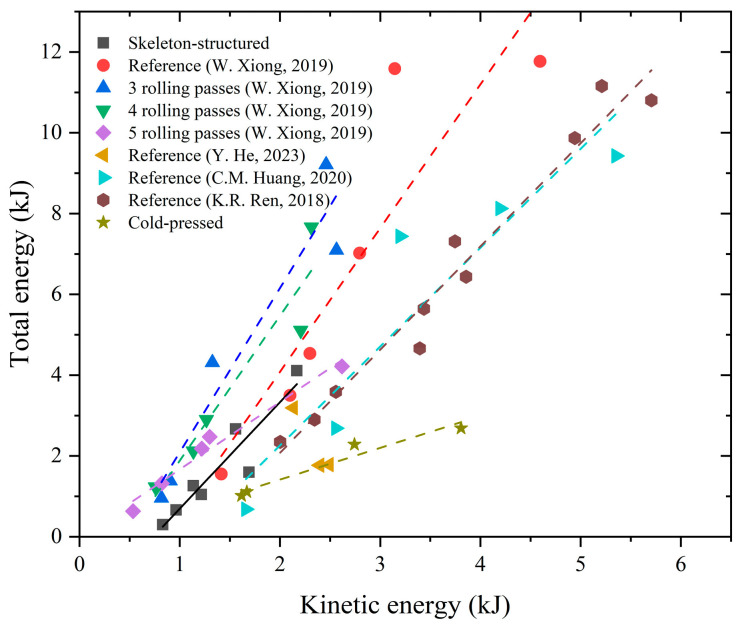
Relationship between kinetic energy and total energy of Al/Ni reactive materials prepared by different processes under impact conditions [36,37,38,44].

From the results of the impact tests, it can be found that both the skeleton-structured Al/Ni reactive material and the cold-pressed Al/Ni reactive material do not easily react under the atmosphere of argon, and the reaction is violent under the atmosphere of air, which also shows that the above two materials need the participation of oxygen to have a violent reaction. Combined with the research results of Huang [38], Graham [39], Iyer [40], Eakins [41], Thadhani [42], Tucker [43], Hu [22], and Ren [44], the two Al/Ni reactive materials prepared in this paper exhibit the characteristics of an aerobic reaction, as do the Al/Ni reactive materials prepared by other processes. At present, many scholars have also carried out a quasi-sealed chamber test of Al/Ni reactive materials and obtained the overpressure and the corresponding total energy test data of Al/Ni reactive materials in the chamber. To compare the reaction characteristics of the Al/Ni reactive material prepared in this paper with those prepared by other processes under impact conditions and then analyze the reaction mechanism of the Al/Ni reactive material under impact conditions, the relationship between the kinetic energy of the fragment and the total energy in the chamber was drawn, as shown in Figure 20. The straight line in the figure is the fitting curve of each data point. The slope of the line represents the ratio of total energy to kinetic energy. The higher the slope, the stronger the reaction of the material and the more energy output.

As shown in Figure 20, in the relationship between kinetic energy and total energy, the performance of Al/Ni reactive materials prepared by different processes is similar; when kinetic energy increases, the total energy released by reactive materials will increase. According to the fitting curves of the energy relationship of various Al/Ni reactive materials shown in the figure, the slope of the skeleton-structured Al/Ni reactive materials is almost the same as that of the hot-pressed Al/Ni reactive materials. The slope of the cold-pressed reactive materials is the lowest, and the slope of the reactive materials prepared by the multiple accumulative rolling process decreases with the increase in rolling times; but they are all higher than that prepared by the cold-press process. The slope of the Al/Ni reactive material prepared by a cold-press process under 850 MPa pressure is the highest.

**Figure 21:**
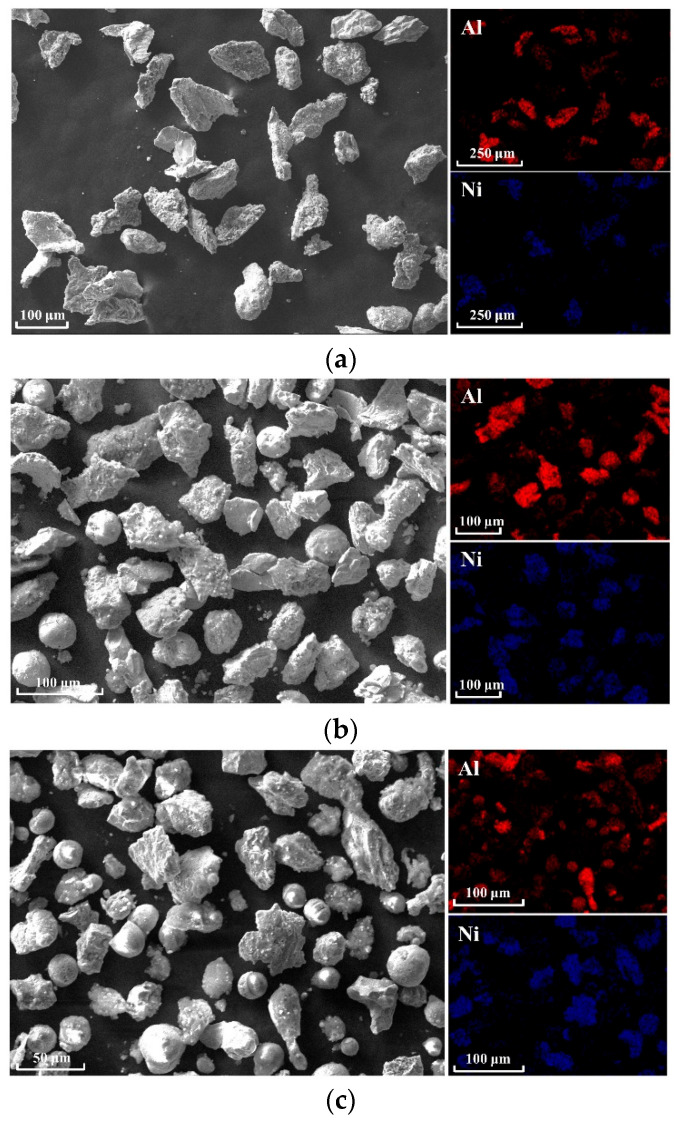
Microstructure and elemental surface distribution of skeleton-structured Al/Ni reactive materials recovered at the impact velocity of 1297 m/s: (**a**) large particles, (**b**) medium particles, and (**c**) small particles.

To verify the mechanism of impact energy release, the recovered products of the experiment in the air environment were analyzed, as shown in Figure 21a. It can be seen from the results of the SEM and EDS tests that in the powder particles with larger particle sizes, the particle shape is irregular, and there are many aluminum and nickel particles. At this time, a small amount of irregular Al/Ni compounds can be found. The irregular shapes of the fragments may be due to insufficient reaction conditions of the fragments after fragmentation, and the reaction cannot be carried out or maintained; a sample of this size is only broken, but no reaction occurs. As shown in Figure 21b, among the particles with medium particle sizes, it can be found that although there are still irregular particles, spherical or spheroid particles appear. There are still a large number of aluminum particles and nickel particles, and a small number of irregular aluminum–nickel compounds exist. As shown in Figure 21c, among the particles with smaller particle sizes, it can be obviously found that a small number of irregular aluminum–nickel compounds exist at this time, and the number of spherical particles increases. Combined with the results of the EDS test, it can be found that spherical particles exist in both elements, indicating that the temperature conditions for melting aluminum and nickel materials can be achieved at this impact velocity. The local temperature is higher than the melting point of nickel (1453 °C), and the particles generated after the impact of the material flow and quickly solidify after melting at high temperatures to form spherical particles.

It is now generally accepted that the fragment temperature increases during the impact process due to impact compression and adiabatic shear. After being subjected to an external force, plastic deformation will occur inside the material, forming a shear zone, and cracks will form and expand in the pores of the material and merge with defects, holes, and other cracks in the material, thus causing fragmentation [45]. Under the impact condition, due to the special structure of the skeleton and the holes and defects existing in the preparation of the material, the shockwave generated by the high-speed impact inside the material will lead to a local impact temperature rise, and the temperature field distribution becomes uneven. In addition, the aluminum–nickel compound generated at the aluminum–nickel interface during the preparation may hinder the contact between aluminum and nickel under such conditions, resulting in a combination reaction between aluminum–nickel, which does not easily occur. The degree of combination reaction is low, and the aluminum–nickel compound produced during the preparation will also be broken after the impact. A small amount of aluminum–nickel compound in the recovered particles may be produced by the reaction between the aluminum–nickel and the compound broken at the aluminum–nickel interface. There are many irregular aluminum and nickel particles in the recovered particles, indicating that the material reaction is insufficient and the reaction degree is low. According to the test and recovery results under different atmospheres, the quasi-static pressure generated by the skeleton-structured Al/Ni reactive material under the impact should be caused by the combustion of some particles to form oxides. At a higher impact velocity, the stronger the impact temperature rise on the sample, the more denser adiabatic shear bands will be generated inside the material, and the more temperature rise will be accumulated. Therefore, particles of different sizes have different temperatures, and smaller particles often have a higher temperature rise, which is also the reason why the number of spherical particles is found at a higher impact velocity.

According to the results of the quasi-static pressure data and the results of the recovered products, it can be found that during the impact condition, the skeleton-structured Al/Ni reactive materials and cold-pressed Al/Ni reactive materials both react more vigorously in the air atmosphere. This shows that the quasi-static pressure of the two materials under the impact condition is caused by a violent oxidation reaction, and the oxidation reaction is the main part of the reaction. In the results of the quasi-static pressure data in this section, it can be found that the reaction degree of the skeleton-structured Al/Ni reactive material is greater than that of the cold-pressed Al/Ni reactive material, and the peak value of the quasi-static pressure is also higher at the same impact velocity. Iyer et al. [40] found in their research that it was difficult to find evidence of a reaction between aluminum–nickel when the sizes of metal particles were reduced under the same impact velocity of flying blades; the smaller the particle size, the more difficult the reaction. It can be considered that the cold-pressed Al/Ni reactive material, because the nickel and aluminum particles are spherical and in a weak bonding state, undergoes impact conditions where, after the adiabatic shear band is formed, cracks occur and expand rapidly, leading to fracture. This leads to less temperature accumulation and lower temperature of the particles formed through fragmentation. Under the premise of oxidation reaction, the particle temperature is low, resulting in a lower degree of oxidation reaction, so that the quasi-static pressure peak of the cold-pressed Al/Ni reactive material under impact conditions is also low. As for the Al/Ni reactive material prepared by scholars [38,44] using the hot-pressing process, particles will form a sintering neck in the initial sintering stage, the grain boundary will be formed at the sintering neck, and the gap between the particles will form pores, which will gradually become smaller as the sintering continues. In the sintering process of the aluminum–nickel powder mixture, aluminum particles are more likely to undergo the changes described above in the sintering stage. This leads to aluminum particles being coated with nickel particles, causing the aluminum particles to combine. This process helps reduce the volume shrinkage of the formed powder, reduce porosity, and enhance its integrity. The aluminum–nickel reactive material prepared by the cold-pressing process under 850 MPa pressure is denser than the Al/Ni reactive material prepared by cold pressing in this paper. The two types of Al/Ni reactive materials have higher shear temperature rises during impact, resulting in higher particle temperatures being generated by fragmentation, so the chip oxidation reaction heat release is higher at a high impact velocity.

Different from the Al/Ni reactive material prepared by powder materials, the Al/Ni reactive material using the accumulative rolling process has a smaller rolling frequency and significantly greater energy release than the powder-pressed material under the same kinetic energy. If the reaction between aluminum and nickel is considered the main reaction under the impact condition, the aluminum and nickel reactive materials prepared by cold-pressing and hot-pressing processes have a larger contact area and are more easily mixed with each other. Then, the energy release performance of the aluminum and nickel reactive materials prepared by these two processes should be stronger than the accumulative rolling process, but the real data performance is the opposite. Therefore, this paper believes that the main reaction of Al/Ni reactive materials under impact conditions is the oxidation reaction. For cylindrical specimens, the failure mode under high-speed impact is mainly composed of spalling failure exceeding the tensile stress of the material, shear failure exceeding the shear stress of the material, and coupling failure. Among them, shear failure is considered the main source of a material’s temperature rise [44]. Before the shear failure of metal materials, the initiation and development of an adiabatic shear zone will occur locally, in which hole defects will appear and continue to increase and expand. Then, cracks will be formed in series, and the material will suffer macroscopic damage, and small particles will be formed. In this case, when the material is destroyed in the adiabatic shear zone, the shear zone reaches a very high temperature, and the broken particles also have a very high temperature. Upon contact with air, there will be a violent oxidation reaction with oxygen. The severity of the reaction depends on the contact area and temperature between the particles and the air. The temperature of broken particles produced by different Al/Ni reactive materials under impact conditions is inconsistent, resulting in inconsistent degrees of chemical reactions and, ultimately, inconsistent results of the energy release effect.

## 6. Conclusions

In this paper, a new type of reactive material based on a skeleton structure was prepared. With nickel used as the skeleton and aluminum as the filling, the mechanical properties of the Al/Ni reactive material were tested, numerical simulations and impact energy release experiments were carried out, and the following conclusions were obtained:

(1) The prepared new reactive material has good compressive properties and certain tensile properties, and the nickel skeleton plays an important supporting role in resisting deformation. The mechanical tests of different strain rates show work hardening and strain rate strengthening effects.
(2) The internal structure of the material was successfully scanned by industrial CT, and the numerical simulation of its mechanical properties under different strain rate compression conditions was carried out based on this. Considering the defects in the actual structure of the material and the presence of aluminum–nickel compounds, the numerical simulation model still needs to be further optimized.
(3) The ballistic impact experiments carried out under an atmosphere of air and argon show that the skeleton-structured Al/Ni reactive material has a certain impact energy release ability, and its overpressure peak value is slightly higher than that of the Al/Ni reactive material prepared by the cold-pressing process, showing better energy release ability. Combined with the impact experiment results and product analysis under argon, it is shown that the energy release of the Al/Ni reactive material mainly depends on the oxidation reaction during the impact process rather than the combination reaction between aluminum and nickel.

In the future, the skeleton structure design of the material could be further optimized to enhance its mechanical properties and improve its energy release capability. Additionally, the changes in microstructure and mechanical properties after different heat treatment processes should be investigated. New metallic elements could also be introduced to design novel composite reactive materials, thereby expanding the application scenarios of this type of material.

## Figures and Tables

**Table 1:** Parameters of Al/Ni reactive materials.

Material	Size (mm)	Al/Ni (wt.%)	Actual Density (g/cm^3^)	TMD
Skeleton-structured Al/Ni reactive material	Φ10 × H9	41.4/58.6	4.16	91%
Cold-pressed Al/Ni reactive material	Φ10 × H9	41.4/58.6	4.38	96%

**Table 2:** SHPB test results of materials under different strain rates.

Strain Rate (s^−1^)	Yield Stress (MPa)	Ultimate Stress (MPa)
700	49.98	91.80
900	46.50	114.79
2300	73.68	178.37
2400	74.37	166.41
2500	75.87	178.80
3100	75.91	204.70
3400	78.23	207.16

**Table 3:** Test data of skeleton-structured and cold-pressed Al/Ni reactive materials under different gas atmospheres.

Material	Atmosphere	Impact Velocity (m/s)	Quasi-Static Pressure Peak (MPa)
Skeleton-structured Al/Ni reactive material	Air	811	0.006
	Air	881	0.013
	Air	982	0.02
	Air	1054	0.025
	Air	1117	0.032
	Air	1201	0.053
	Air	1370	0.081
	Argon	898	0.017
	Argon	986	0.012
	Argon	1118	0.016
	Argon	1216	0.019
	Argon	1287	0.018
	Argon	1341	0.016
Cold-pressed Al/Ni reactive material	Air	935	0.02
	Air	953	0.022
	Air	1215	0.045
	Air	1403	0.053
	Argon	870	0.012
	Argon	1110	0.015
	Argon	1232	0.026
	Argon	1248	0.023
	Argon	1331	0.018
	Argon	1328	0.019
	Argon	1376	0.021

**Table 4:** Summary of numerical simulation results and real test results.

Type of Results	Yield Stress (MPa)	Yield Strain (%)	Ultimate Stress (MPa)	Plastic Strain (%)
Average result of tests	68.2	3.90	149.6	17.80
Numerical simulation results	42.6	0.55	150.9	14.40

**Table 5:** Results of numerical simulation data.

Strain Rate (s^−1^)	Yield Stress (MPa)	Ultimate Stress (MPa)
1000	47.6 ± 3	112.48
1300		121.70
1600		130.86
2000		140.27
2900		144.19
3600		147.64

**Table 6:** Parameters of skeleton-structured Al/Ni reactive materials.

Actual Density (g/cm^3^)	Heat of Reaction *E* (kJ/g)
4.16	16.8

**Table 7:** Energy release efficiency of skeleton-structured Al/Ni reactive materials at different impact velocities.

Impact Velocity (m/s)	Quasi-Static Pressure Peak (10^−1^ MPa)	Total Energy (J)	25% Residual Kinetic Energy (J)	Energy Difference (J)	Energy Release Efficiency (%)
811	0.05792	289.6	204.49	85.11	0.18
881	0.13158	657.9	238.43	419.47	0.88
982	0.20392	1019.6	299.66	719.94	1.50
1054	0.25167	1258.35	282.49	975.86	2.46
1117	0.31879	1593.95	420.36	1173.59	2.32
1201	0.52974	2648.7	386.10	2262.60	5.54
1370	0.81514	4075.7	540.63	3535.07	8.22

## Data Availability

The original contributions presented in this study are included in the article; further inquiries can be directed to the corresponding authors.
